# Superficial Vein Thrombosis in Obese Patients

**DOI:** 10.3390/jcm14145024

**Published:** 2025-07-16

**Authors:** Lucía Ordieres-Ortega, Rubén Alonso-Beato, Tatiana Pire-García, Sergio Moragón-Ledesma, Marina López-Rubio, Marta-Olimpia Lago-Rodríguez, Luis Antonio Alvarez-Sala Walther, Francisco Galeano-Valle, Pablo Demelo-Rodríguez

**Affiliations:** 1 Venous Thromboembolism Unit, Internal Medicine Department, General University Hospital Gregorio Marañón, 28007 Madrid, Spain; lucia.ordieres@salud.madrid.org (L.O.-O.); marinalopezrubio@outlook.com (M.L.-R.); pbdemelo@hotmail.com (P.D.-R.); 2 School of Medicine, Universidad Complutense de Madrid, 28040 Madrid, Spain; 3 Instituto de Investigación Sanitaria Gregorio Marañón, 28007 Madrid, Spain

**Keywords:** fondaparinux, low molecular weight heparin, superficial vein thrombosis, recurrences, obesity

## Abstract

**Background:** The optimal anticoagulation strategy for obese patients with superficial vein thrombosis (SVT) remains unclear. This study evaluates the impact of obesity on anticoagulation patterns and clinical outcomes in patients with lower limb SVT. **Methods:** We conducted a prospective observational study including consecutive patients with SVT in a tertiary hospital from 2014 to 2024. Patients with SVT ≥ 5 cm in length and ≥3 cm from the saphenofemoral junction were included. Obese (BMI ≥ 30) and non-obese (BMI < 30) patients were compared. Patients were followed for one year. Outcomes were assessed at 90 and 365 days. The primary outcomes were venous thromboembolism (VTE) recurrence (SVT, deep vein thrombosis [DVT], or pulmonary embolism [PE]). The secondary outcomes were major bleeding and all-cause mortality. **Results:** Of 136 patients, 58 (42.6%) were obese. Both groups had similar baseline characteristics, except for younger age and higher smoking prevalence in obese patients. Most patients received anticoagulation (91.9%), primarily a prophylactic dose of low molecular weight heparin or a prophylactic dose of fondaparinux. No significant differences were found in VTE recurrence at 90 or 365 days (*p* = 0.505), and no major bleeding events occurred. Female sex was associated with a higher risk of VTE recurrence (OR 4.33, 95% CI 1.17–15.98, *p* = 0.028), but obesity did not influence outcomes. **Conclusions:** Obesity was not associated with increased VTE recurrence in patients with lower limb SVT. No major bleeding events were observed. These findings suggest that standard anticoagulation regimens may be appropriate for obese patients with SVT, but further studies are needed to confirm these results.

## 1. Introduction

Superficial vein thrombosis (SVT) of the lower limbs was traditionally called thrombophlebitis. This name has long been considered inadequate since infection is not a central part of this disease [1]. SVT has been considered a relatively mild and benign condition and has received less attention than other more serious thrombotic events. However, recent evidence highlights that SVT has the potential to progress to deep vein thrombosis (DVT) or pulmonary embolism (PE), with reported rates of up to 53 and 10% of cases, respectively [2]. The epidemiology of SVT underscores its significance, with an estimated incidence ranging from 0.3 to 1.5 per 1000 person-years [3]. This figure increases notably among older adults, reaching up to 2.92 per 1000 person-years [2]. In light of these numbers, comprehensive diagnostic imaging is essential in patients with suspected SVT of the lower limbs. In these patients, a whole-leg ultrasound is recommended to assess thrombus extension and rule out concomitant DVT [4]. Several risk factors have been identified for the development of SVT. These include the presence of varicose veins, which is the most frequently associated condition, and other conditions common to venous thrombosis, such as a personal or family history of venous thromboembolism (VTE), cancer, inherited thrombophilia, pregnancy, and hormonal therapies [2]. Unlike other types of VTE, varicose veins are the main risk factor for lower limb SVT and are found in 90% of cases [3].

Although SVT is generally less life-threatening than DVT or PE, recurrence remains a significant concern. The Prospective Observational Superficial Thrombophlebitis (POST) study [5] was a large, international, prospective observational study designed to evaluate the natural history, risk factors, and management of SVT in routine clinical practice. Conducted across multiple centers, it included over 800 patients with confirmed SVT of the lower limbs. One of its key findings was that anticoagulation significantly reduced the risk of thromboembolic complications compared to conservative management. Among 600 patients without DVT or PE at inclusion, 10.2% developed thromboembolic complications at 3 months, either PE, DVT, progression of SVT, or recurrent SVT. Risk factors for complications at 3 months were male sex, history of DVT or PE, previous cancer, and absence of varicose veins.

Given the risk of concomitant or progressing thromboembolism, guidelines [4] now advocate for whole-leg duplex ultrasonography in patients with suspected SVT. This examination not only confirms the diagnosis but also detects associated silent DVT, assesses extension toward the saphenofemoral/saphenopopliteal junctions, and provides baseline information that is crucial for management decisions.

Recurrence rates for SVT are comparable to those of other venous thrombotic events, although most recurrences present as SVT rather than DVT or PE [6]. Given this, effective management is crucial to prevent complications. Current guidelines [4] recommend anticoagulation with fondaparinux 2.5 mg once daily, prophylactic doses of low molecular weight heparin (LMWH) or rivaroxaban 10 mg once daily for 45 days for patients with lower limb SVT located ≥ 3 cm from the saphenofemoral junction and extending ≥ 5 cm in length [2]. A shorter duration of treatment has been associated with a higher risk of recurrent events [4]. A recent meta-analysis has shown that short-term treatment protocols (14 days or less) were associated with the highest rates of VTE complications [7], about 59.7 events per 100 patient-years.

Obesity is defined as the presence of excessive fat accumulation that poses a health risk. A body mass index (BMI) ≥ 30 kg/m^2^ is commonly used as the diagnostic threshold. Obesity is considered a minor risk factor for a first episode of VTE, but its impact on VTE recurrence remains uncertain. In a systematic review, three studies found a significant association between obesity and VTE recurrence in women, but not in men, after a first episode of unprovoked VTE. However, obesity was not associated with recurrence in patients with distal DVT. The review concluded that current evidence provides conflicting results regarding whether obesity increases the risk of VTE recurrence, although it does not specifically address patients with SVT [8].

In patients with a BMI greater than 30, data regarding optimal anticoagulation strategies and clinical outcomes in the context of isolated SVT remain scarce. For instance, the optimal anticoagulant choice and dosing in obesity is unclear. Most trials, while including overweight patients, did not stratify by BMI. Additionally, few studies provide robust follow-up beyond three months. Finally, clinical trials often exclude patients with comorbidities such as cancer or prior VTE, thus lacking real-world applicability. This highlights a key gap in current SVT management, particularly for obese patients, who may present with altered pharmacodynamics and increased thrombosis risk.

Our study aims to address this need by analyzing anticoagulation usage, dosing, efficacy, and clinical outcomes in a contemporary cohort of obese (BMI ≥ 30) patients with isolated SVT managed within a VTE unit. By integrating factors such as demographics, comorbid conditions, treatment modalities (DOAC vs. LMWH vs. fondaparinux), and follow-up imaging, we aim to offer guidance for this underserved but clinically important subgroup.

## 2. Materials and Methods

### 2.1. Type of Study

This prospective observational study utilized data from a tertiary Spanish hospital registry. Patients with a diagnosis of acute SVT of lower limbs confirmed by objective imaging (doppler ultrasound) between January 2014 and October 2024 were consecutively enrolled. All patients provided informed consent in accordance with local ethics committee requirements.

Ultrasounds were performed by clinicians with at least 50 h of accredited training, over 400 compression ultrasounds performed, and frequent use of the technique in the VTE clinic setting.

### 2.2. Patients and Exclusion Criteria

We included patients enrolled between January 2014 and October 2024 who were diagnosed with SVT, either in the Emergency Department or during hospitalization.

Patients were eligible if they met the following criteria: (1) age > 18 years; and (2) imaging-confirmed SVT of lower limbs extending ≥ 5 cm in length and located ≥ 3 cm from the saphenofemoral junction. Exclusion criteria included: (1) patients diagnosed with SVT extending < 5 cm; (2) patients diagnosed with SVT located < 3 cm from the saphenofemoral junction; (3) patients diagnosed with SVT and concomitant DVT or PE; and (4) patients with a previous indication for therapeutic-dose anticoagulation.

### 2.3. Variables

Baseline data included demographic characteristics (sex, age, weight, BMI) and clinical characteristics (smoking, hypertension, diabetes, dyslipidemia, cancer, thrombophilia testing, presence of varicose veins, personal or family history of VTE, and previous saphenectomy). Clinical characteristics also included previous anticoagulation therapy and management of a present SVT event. Thrombophilia testing was performed according to clinical judgment.

### 2.4. Study Outcomes

Obese patients (defined by a BMI ≥ 30) were compared to non-obese patients (BMI < 30). Patients were followed for one year. Outcomes were assessed at 90 and 365 days. The primary outcome was VTE recurrence (PE, DVT, or SVT). The secondary outcomes were major bleeding and all-cause mortality.

PE was defined as a filling defect on a pulmonary artery computerized tomography (CT) or a ventilation/perfusion mismatch on lung scintigraphy. DVT was confirmed by duplex ultrasound imaging. Major bleeding was defined as acute, clinically overt bleeding associated with at least one of the following: a hemoglobin drop of ≥2 g/dL, transfusion of ≥2 units of red blood cells, bleeding at a critical site (intracranial, intraspinal, intraocular, pericardial, intraarticular, intramuscular with compartment syndrome, or retroperitoneal), bleeding requiring surgical intervention, or fatal bleeding [9].

Therapeutic options included prophylactic LMWH (adjusted to body weight), prophylactic fondaparinux (2.5 mg once daily), or DOACs (rivaroxaban 10 mg once daily).

### 2.5. Ethics and Risks

This study was conducted in full accordance with internationally recognized ethical standards for research involving human participants. Specifically, it adhered to international ethical guidelines for research involving human participants, following the latest revision of the Declaration of Helsinki. In addition, the study complied with the International Council for Harmonisation’s Good Clinical Practice (ICH-GCP) guidelines, which provide a unified standard to ensure the integrity of data and the protection of participants in clinical research. All applicable national and institutional regulations governing human subject research were also strictly followed.

Prior to its initiation, the study protocol underwent rigorous ethical review and received formal approval from the institutional Ethics Committee of the participating center (protocol code 23/2022, 19 December 2022).

All individuals who participated in the study provided informed and voluntary consent. Furthermore, the confidentiality of all collected data was rigorously maintained. Personal identifiers were removed or anonymized, and all data were stored securely in accordance with data protection regulations to ensure privacy and limit access to authorized personnel only.

### 2.6. Statistical Analysis

Qualitative variables were presented as frequencies and percentages for qualitative variables. Age, as a non-normally distributed variable, was reported as the median and interquartile range (IQR) (25th and 75th percentiles), using the Shapiro–Francia test for normality and Levene’s test for variance homogeneity. Associations between categorical variables were evaluated using the chi-square or Fisher’s exact test, as appropriate. Continuous variables were compared using the Mann–Whitney U test. A *p*-value < 0.05 was considered statistically significant. Logistic regression analyses were performed to assess associations between different variables, estimating odds ratios (OR) and 95% confidence intervals (CI). Statistical analyses were performed using IBM SPSS Statistics for Windows^®^, Version 25 (IBM Corp, Armonk, NY, USA).

## 3. Results

A total of 237 patients were identified, of whom three were excluded due to concomitant PE or DVT. Among the remaining 234 patients, 80 had thrombi located < 3 cm from the saphenofemoral junction, and 18 had SVT extending < 5 cm in length. The final study population consisted of 136 patients, including 58 with a BMI ≥ 30 and 78 with a BMI < 30. Patients with a BMI ≥ 30 were younger and more frequently smokers than those with a BMI < 30. Otherwise, there were no significant differences in the presence of varicose veins, a personal or family history of VTE, or cardiovascular risk factors. Thrombophilia testing was performed in 14 patients, of which 10 were non-obese and four were obese (*p* = 0.024). Testing was positive in five of the obese patients, but there were no positive results in the non-obese population. Among the nine patients with cancer, three were obese and six non-obese. The baseline characteristics are summarized in Table 1.

The majority of patients received anticoagulation. More than half were treated with prophylactic LMWH (55.17% in obese and 61.45% in non-obese patients, *p* = 0.456). Approximately one-third received 2.5 mg of fondaparinux daily in both groups (36.21% in obese and 30.77% in non-obese patients, *p* = 0.505). The median duration of anticoagulant therapy was 45 (IQR 44–132) days in obese patients and 45 (IQR 43–116) days in non-obese patients (*p* = 0.58).

The incidence of VTE recurrence, all-cause mortality, and major bleeding was similar in both groups. No major bleeding events were observed. The general outcomes are summarized in Table 2. In the univariate analysis, obesity was not associated with an increased risk of VTE recurrence at 90 or 365 days, either as SVT (OR 2.07 [95% CI 0.33–12.82] and OR 1.65 [95% CI 0.52–5.91], respectively) or as another type of venous thrombosis (OR 2.07 [95% CI 0.33–12.82] and OR 1.40 [95% CI 0.492–3.98]). Similarly, cancer was not associated with an increased risk of VTE recurrence (OR 0.94; 95% CI 0.19–4.81).

Among the 16 patients with VTE recurrence, 13 were female and three were male (crude OR 4.33 [95% CI 1.17–15.98], *p* = 0.028). Regarding obesity, no recurrence events were observed in non-obese males, while eight non-obese females experienced recurrence (Fisher’s exact test, *p* = 0.001). In obese patients, no significant differences were found between men and women (OR 0.94 [95% CI 0.20–4.34]). All VTE recurrences occurred after anticoagulation withdrawal. Univariate analysis did not show a significant association between obesity and all-cause mortality at either 90 or 365 days (*p* = 0.883 in both cases). A multivariate analysis including age, smoking habit, and thrombophilia testing did not show any significant results. The outcomes are summarized in Table 2 and Table 3.

## 4. Discussion

There is limited evidence on the optimal anticoagulation strategy for obese patients with SVT, highlighting a significant gap in current knowledge. Obesity is a recognized risk factor for VTE, including SVT. Nevertheless, most clinical trials evaluating anticoagulation in SVT have not stratified results by body weight or body mass index (BMI), making it difficult to determine whether dosage adjustments are necessary or if alternative agents may offer improved outcomes. As a result, clinicians often rely on extrapolation from studies in DVT or expert opinion when managing obese patients with SVT.

Our study found that those with a BMI over 30 were younger, more often smokers, and more frequently women compared to patients with SVT and a BMI under 30, similarly to previous research [3]. Treatment strategies and event rates were similar between both groups.

Among patients with SVT in non-varicose veins, previous research has reported a high prevalence of thrombophilia [10]. The incidence of thrombophilia might be higher in patients with SVT [2]. Milio et al. [11] reported that the prevalence of all these gene mutations was higher in patients with SVT extending to the deep venous system than those without extension, regardless of the presence or absence of varicose veins. In our population, patients with DVT were excluded. In any case, the incidence of positive thrombophilia was higher in the non-obese group. It is important to note that thrombophilia testing was performed in only 14 patients (10.29% of the study population), in line with recent, more restrictive evidence on when to perform such testing [12]. Recent guidelines [13] do not include thrombophilia testing in patients with SVT, and general evidence supports limiting such testing to situations where the results would alter clinical management. Interestingly, thrombophilia testing was performed more frequently in obese patients, with this difference reaching statistical significance. To better understand the role and clinical utility of thrombophilia testing specifically in obese individuals with SVT, studies involving a larger and more diverse patient population are warranted.

Previous studies have suggested an association between male sex and an increased risk of VTE recurrence after a first episode of SVT [3,5]. A meta-analysis identified male sex, older age, history of VTE, absence of varicose veins, and cancer as factors associated with a worse prognosis [14]. In our study, we observed no significant sex differences between the two subgroups in terms of baseline characteristics. However, female sex was associated with a higher risk of VTE recurrence within the non-obese population. Interestingly, this association was not observed among obese patients, suggesting that obesity may modify or mitigate the influence of sex on VTE recurrence risk, perhaps due to the higher mean age of the non-obese population or the influence of obesity as a prothrombotic factor that could override the sex-related hormonal effects. Furthermore, consistent with previous studies [15], the presence of cancer did not appear to significantly increase the risk of VTE recurrence in our cohort.

A history of SVT may correlate with a higher risk of future recurrence of VTE as PE or DVT [16]. In our population, eight out of 58 of the obese patients (13.8%) presented a VTE recurrence. Interestingly, only one patient suffered DVT, while the remaining recurrences occurred in the form of SVT. When comparing recurrence rates between obese and non-obese patients, no statistically significant differences were observed, suggesting that obesity alone may not be a distinguishing factor for VTE recurrence in this population.

The CALISTO trial (Comparison of Arixtra in Lower Limb Superficial Vein Thrombosis with Placebo) [17], published in 2010, was a landmark, multicenter, randomized, double-blind, placebo-controlled study that significantly influenced the management of superficial vein thrombosis (SVT). It enrolled 3002 patients with acute, symptomatic SVT of the lower limbs (≥5 cm in length), but without concomitant deep vein thrombosis (DVT) or symptomatic pulmonary embolism (PE). Patients were randomized to receive either 2.5 mg of fondaparinux once daily for 45 days or a placebo, with a follow-up period of 77 days. The trial demonstrated a significant reduction in the composite primary endpoint—which included death from any cause, symptomatic PE, symptomatic DVT, symptomatic extension to the saphenofemoral junction, or symptomatic recurrence of SVT—in the fondaparinux group (0.9%) compared to the placebo (5.9%), representing an 85% relative risk reduction (*p* < 0.001). Importantly, the safety profile of fondaparinux was favorable, with no significant increase in major bleeding events.

Among the CALISTO population, 574 patients (approximately 19%) had a body mass index (BMI) > 30, meeting the criteria for obesity. The trial did not report significant differences in treatment efficacy or adverse events within this subgroup compared to the overall population, though detailed subgroup analyses based on BMI were limited. As such, while obese patients were represented, the study was not powered to specifically evaluate differences in outcomes between obese and non-obese individuals with SVT.

Subsequently, Cosmi et al. conducted the STEFLUX trial (Superficial ThromboEmbolism and Fluxum) [15], a randomized, double-blind, multicenter study designed to evaluate the efficacy and safety of parnaparin, a low molecular weight heparin (LMWH), in the treatment of superficial vein thrombosis (SVT). The trial included 427 patients with acute, symptomatic SVT of the lower limbs and randomized them to receive either a prophylactic dose (4250 IU/day) or a therapeutic/intermediate dose (8500 IU/day) of parnaparin for 10 days, followed by a standardized tapering regimen over the subsequent 20 days. The primary efficacy endpoint was the composite of symptomatic extension or recurrence of SVT, symptomatic DVT, symptomatic PE, or all-cause mortality at 33 days. The results showed that both regimens were effective and well-tolerated, with a low incidence of thromboembolic complications. There was no statistically significant difference in efficacy between the two dosing strategies. Nonetheless, there was a numerical trend favoring the intermediate dose, suggesting potential added benefit in patients at higher risk of progression. The safety profile was favorable in both arms, with no major bleeding events reported. This study did not focus specifically on obese patients, and no subgroup analysis based on BMI was reported.

Finally, the SURPRISE trial (Superficial Vein Thrombosis Treated for 45 Days With Rivaroxaban Versus Fondaparinux) [18] was another important randomized, open-label, non-inferiority trial aimed at comparing 10 mg of oral rivaroxaban once daily with 2.5 mg of fondaparinux once daily, both administered for 45 days, in patients with symptomatic lower limb SVT. A total of 472 patients were included. The primary efficacy outcome—a composite of symptomatic DVT, PE, extension or recurrence of SVT, and VTE-related death—occurred in 3.3% of the fondaparinux group and 2.1% of the rivaroxaban group, demonstrating non-inferiority of rivaroxaban. Major bleeding events were rare and comparable between groups.

The mean BMI in the SURPRISE trial was 28.7 (IQR 25.8–33.0) in the fondaparinux arm and 29.0 (IQR 25.8–33.4) in the rivaroxaban arm, indicating that a substantial proportion of patients were overweight or obese. However, the study did not report the exact number of patients with BMI ≥ 30, and no stratified analysis by BMI category was provided. As a result, while the data suggest that these agents are likely effective across a range of BMI values, firm conclusions about their comparative effectiveness in obese patients remain uncertain.

Based on the findings of these and other studies, current international guidelines [19] recommend 2.5 mg of fondaparinux daily for 45 days as the first-line therapy for SVT involving the lower limbs, particularly when the thrombus is ≥5 cm in length or close to the deep venous system. Alternative options include prophylactic doses of LMWH and 10 mg of rivaroxaban daily, administered for the same duration, especially in cases where fondaparinux is contraindicated or unavailable. This is the rationale followed in this study.

In our study population, the number of patients receiving direct oral anticoagulants (DOACs), such as rivaroxaban, was limited. This low usage rate precluded a meaningful subgroup analysis based on DOAC treatment, which is particularly relevant given the increasing adoption of oral agents in clinical practice. Further real-world studies focusing on obese patients with SVT and comparing outcomes across different anticoagulation strategies are warranted to guide individualized treatment decisions in this growing and often undertreated subgroup.

The existing literature suggests that older age, male sex, a personal history of VTE, active cancer, and the absence of varicose veins might be potential predictors of a higher risk of clot propagation in patients with SVT. Notably, obesity was not identified as a contributing risk factor in this study. In our cohort, however, obese patients were significantly younger than their non-obese counterparts. According to current evidence, this demographic characteristic would typically be associated with a lower thrombotic risk, which may help explain this finding. Moreover, the so-called “obesity paradox”, although controversial, has been previously described. This paradox suggests that obese patients may experience better outcomes following a VTE event compared to non-obese patients, possibly due to earlier diagnosis, higher cardiac output, or selection bias. Nonetheless, this paradox has not been consistently observed and may be attributable to confounding or other biases. Additionally, the recent literature [20] suggests that body fat percentage and waist circumference may be superior markers of thrombotic risk compared to BMI. Future studies should consider measures of body fat percentage and distribution to improve risk stratification.

Nevertheless, the unique risk profile of obese individuals with SVT remains insufficiently defined. Ongoing studies may help expand the current understanding and guide more tailored management strategies for this patient subgroup [2]. Future research is already aiming to establish prognostic prediction models for relevant clinical outcomes in SVT [21]. Ongoing registries and studies such as the START2 Registry (Survey on Anticoagulated Patients) and the METRO study (MEsoglycan for Secondary Prevention of Superficial Vein Thrombosis; ClinicalTrials.gov Identifier: NCT 03428711) may help expand the current evidence base regarding optimal management strategies for obese patients with SVT [2].

The main limitations of our study include its relatively small sample size and its single-center design, which may limit the generalizability of our findings. Furthermore, the small subgroup of patients receiving DOACs prevented further analysis of this population. However, to the best of our knowledge, this is the first study specifically assessing SVT outcomes in obese patients. An additional strength is that all patients were managed within a dedicated VTE unit, ensuring a standardized approach to both diagnosis and treatment. Moreover, our study included some patients with a prior history of VTE, who are often excluded from clinical trials, thus adding to the real-world relevance and applicability of our findings. Upcoming research should include patients undertaking DOACs and a larger population, perhaps with a multicenter design.

## 5. Conclusions

In conclusion, in our study, obesity was not associated with an increased risk of VTE recurrence in patients with lower limb SVT. Notably, no major bleeding events were observed during follow-up. These findings suggest that current anticoagulation strategies for SVT may be appropriate regardless of BMI, but further research is needed to confirm these results.

## Figures and Tables

**Table 1:** Baseline characteristics and treatment of the patient populations, comparing obese and non-obese patients. BMI = body mass index; IQR = interquartile range; LMWH = low molecular weight heparin; SVT = superficial vein thrombosis; VTE = venous thromboembolism.

*n* = 136	Obesity (*n* = 58)	Non-Obesity (*n* = 78)	*p*-Value
Male sex (*n*, %)	21 (36.21%)	42 (53.85%)	0.041
Age (median, IQR)	54 (46–66)	68 (50–76)	0.001
Weight (median, IQR)	92.5 (78–107)	73 (67–78)	<0.001
BMI (median, IQR)	34.48 (32.04–37.46)	26.11 (24.16–27.88)	<0.001
Smoking (*n*, %)	17 (29.31%)	11 (14.10%)	0.030
Hypertension (*n*, %)	24 (41.38%)	31 (39.74%)	0.848
Diabetes (*n*, %)	8 (13.79%)	12 (15.38%)	1
Dyslipidemia (*n*, %)	12 (20.69%)	23 (29.49%)	0.246
Cancer (*n*, %)	3 (5.17%)	6 (10.71%)	0.732
Presence of varicose veins (*n*, %)	45 (77.59%)	53 (67.95%)	0.215
Personal history of VTE (*n*, %)	10 (17.24%)	10 (12.85%)	0.476
Family history of VTE (*n*, %)	8 (18.60%)	6 (10.71%)	0.264
Saphenectomy (*n*, %)	13 (29.55%)	12 (22.64%)	0.439
Positive thrombophilia	4 (9.30%)	0 (0%)	-
Treatment			
Prophylactic LMWH	32 (55.17%)	48 (61.45%)	0.456
Prophylactic fondaparinux	21 (36.21%)	24 (30.77%)	0.505
Other	5 (8.62%)	6 (7.78%)	0.477

**Table 2:** Outcomes of the univariate analysis for patient populations, comparing obese and non-obese patients. CI = confidence interval; OR = odds ratio; SVT = superficial vein thrombosis; VTE = venous thromboembolism.

Outcomes			
*n* = 136	Obesity (*n* = 58)	Non-Obesity (*n* = 78)	OR (95% CI)
VTE Recurrence (*n*, %)			
Outcomes at 90 days	3 (5.17%)	2 (2.56%)	2.07 (CI 95% 0.33–12.82)
Outcomes at 365 days	8 (13.79%)	8 (10.26%)	1.40 (CI 95% 0.492–3.98)
Recurrence as SVT (*n*, %)			
Outcomes at 90 days	3 (5.17%)	2 (2.56%)	2.07 (0.33–12.82)
Outcomes at 365 days	7 (12.07%)	6 (7.59%)	1.65 (0.52–5.91)
Major Bleeding (*n*, %)			
Outcomes at 90 days	0 (0%)	0 (0%)	-
Outcomes at 365 days	0 (0%)	0 (0%)	-
All-cause Mortality (*n*, %)			
Outcomes at 90 days	0 (0%)	0 (0%)	-
Outcomes at 365 days	0 (0%)	2 (2.56%)	-

**Table 3:** Multivariate analysis adjusting for age, smoking habit, and thrombophilia. OR = *odds ratio*, CI = confidence interval.

Outcomes *n* = 136	OR (95% CI)
**VTE Recurrence (*n*, %)**	
At 90 days	1.77 (0.25–12.23)
At 365 days	1.10 (0.34–3.50)
**Recurrence as SVT (*n*, %)**	
At 90 days	1.77 (0.25–12.23)
At 365 days	1.34 (0.38–4.73)
**Major Bleeding (*n*, %)**	
At 90 days	-
At 365 days	-
**All-cause Mortality (*n*, %)**	
At 90 days	-
At 365 days	-

## Data Availability

Data are unavailable due to privacy or ethical restrictions.

## Abbreviations

The following abbreviations are used in this manuscript:

SVT	Superficial Vein Thrombosis
DVT	Deep Vein Thrombosis
PE	Pulmonary Embolism
VTE	Venous Thromboembolism
LMWH	Low Molecular Weight Heparin
BMI	Body Mass Index
CT	Computerized Tomography
IQR	Interquartile Range
OR	Odds Ratios
CI	Confidence Intervals
DOACs	Direct Oral Anticoagulants
